# THE HIGH PREVALENCE OF OVER-THE-COUNTER MEDICATION MISUSE IN OLDER ADULTS: RESPONSE TO THE CHPA-GSA NATIONAL SUMMIT

**DOI:** 10.1093/geroni/igad104.3645

**Published:** 2023-12-21

**Authors:** Jason Chladek, Aaron Gilson, Jamie Stone, Elin Lehnbom, Emily Hoffins, Maria Berbakov, Jukrin Moon, Michelle Chui

**Affiliations:** University of Wisconsin-Madison School of Pharmacy, Madison, Wisconsin, United States; University of Wisconsin-Madison, Madison, Wisconsin, United States; University of Wisconsin-Madison, Madison, Wisconsin, United States; UiT The Arctic University of Norway, Tromsø, Troms, Norway; University of Wisconsin-Madison, Madison, Wisconsin, United States; University of Wisconsin-Madison, Madison, Wisconsin, United States; University of Wisconsin-Madison School of Pharmacy, Madison, Wisconsin, United States; University of Wisconsin-Madison, Madison, Wisconsin, United States

## Abstract

Older adults (65+) are the largest consumer of over-the-counter (OTC) medications and disproportionately prone to adverse drug events (ADEs). Despite perceptions that OTCs are generally safe, OTC misuse can lead to ADEs. However, as identified through the Consumer Health Products Association (CHPA) and Gerontological Society of America (GSA) National Summit, little is known about OTC misuse in older adults. This is the first muti-site study to prospectively quantify how older adults select OTCs in community pharmacies where products are typically purchased. Older adults (n=144) were recruited from 10 community pharmacies. Participants were given hypothetical symptoms and asked to select one or more OTCs. After selection, they were asked to report how they would use the product, their home medications, and their health conditions. OTC selections were evaluated for the following misuse categories: 1) drug-age (Beers Criteria), 2) drug-drug (interactions with home medications), 3) drug-disease (health conditions), and 4) drug-label (product’s indications and directions). At least one type of misuse was identified in 114 (79%) older adults, with 57 (40%) demonstrating misuse in >1 category. Overall, 363 total instances of misuse were identified (x̄=3.18 per participant). There was high prevalence of drug-drug and drug-label misuse, as 107 (74%) demonstrated misuse in one or both categories. The results highlight OTC misuse frequency and complexity in older adults. Knowing older adults’ OTC misuse prevalence is critical for understanding medication-associated risks and developing effective interventions. Importantly, these results are a first step toward addressing the research gaps identified by CHPA and GSA.

